# Remote Robotic Technology in Minimally Invasive Spinal Surgery: A Comprehensive Literature Review

**DOI:** 10.1155/ijta/6131682

**Published:** 2025-08-06

**Authors:** Aliasghar Karimi, Pegah Pedramfard, Habib Zakeri, Behnam Rahnama, Mohammad Radmehr, Saba Moalemi, Leala Montazeri

**Affiliations:** Research Center for Neuromodulation and Pain, Shiraz University of Medical Sciences, Shiraz, Iran

**Keywords:** remote surgery, spine, telerobotics, telesurgery

## Abstract

Telerobotic spinal surgery (TSS) has emerged as a transformative approach for spinal interventions that combines robotics and telecommunication to enable remote surgery with enhanced precision and accessibility. This technology can potentially address the challenges of complex spinal conditions by providing minimally invasive solutions and delivering specialized surgical expertise across geographical boundaries. However, technical complexities related to communication and latency, haptic feedback, and system integration need to be addressed. Ethical and regulatory considerations also require careful navigation. Despite these challenges, the limited number of available clinical studies demonstrates the feasibility and efficacy of TSS, particularly with the use of 5G technology. Further research and clinical trials are needed to fully evaluate and validate the outcomes and effectiveness of TSS. With ongoing efforts in research and development, TSS has the potential to revolutionize spine surgery and improve patient care. In this review, we present the role and the challenges of TSS in spinal surgery.

## 1. Introduction

In recent years, the convergence of robotics, telecommunication, and medical science has given rise to a transformative approach in the field of spinal surgery, telerobotic spinal surgery (TSS). This cutting-edge technology has ushered in a new era of precision, accessibility, and patient care in the realm of spine interventions. By merging the capabilities of robotics with the potential of telecommunication, TSS has emerged as a promising field, offering innovative solutions to some of the most intricate challenges in spinal healthcare [1, 2].

The spine, a complex and delicate structure central to human movement and stability, presents unique surgical demands. Conditions affecting the spine, ranging from degenerative disorders to traumatic injuries, necessitate precise and often minimally invasive surgical approaches. TSS addresses these demands by enabling skilled surgeons to operate remotely with unprecedented precision, even across vast geographical distances [3]. This remarkable fusion of robotics and telecommunication has the potential to transcend geographical boundaries, making specialized surgical expertise available to patients who were previously constrained by geographical limitations [4].

The advent of TSS is particularly timely, given the global healthcare landscape's growing demand for high-quality and accessible care. This technology empowers surgeons with enhanced visualization, superior dexterity, and the ability to perform intricate maneuvers through minimal incisions. These advantages can translate into reduced patient trauma, shorter recovery times, and improved surgical outcomes [5].

In this review, we embark on an exploration of TSS and will delve into the intricate technical aspects, review the innovative approaches that have propelled the discipline forward, and evaluate the impact of TSS on patient care. Additionally, we will discuss the challenges that must be overcome and the ethical considerations that warrant close attention as this technology evolves.

For this aim, we reviewed the studies that have implemented TSS in live human subjects with the operating surgeon located remotely from the patient. We did a thorough Google Scholar search in English literature, using the keywords “telerobotic,” “spine,” “remote,” and “telesurgery.” The six-item version of SANRA was followed in this narrative review.

## 2. Historical Perspective of Telesurgery

**Figure 1:**
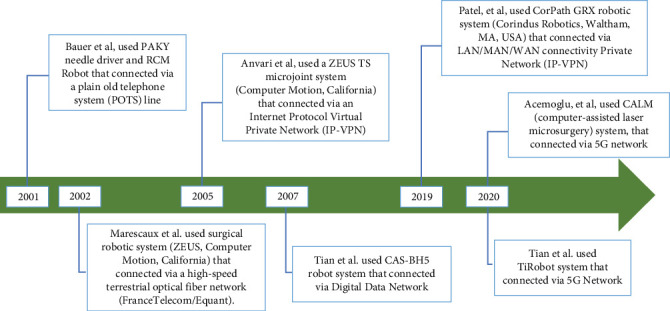
Notable breakthroughs of telesurgery.

In Figure 1, we have depicted the milestones in telesurgery in a temporal manner. Bauer et al. conducted a study of remote renal access surgery in 2001. Using a PAKY (percutaneous access of the kidney) robot connected to a plain old telephone service (POTS) line, the surgeon in Baltimore, Maryland, was able to successfully perform a percutaneous access procedure on a patient in Rome, Italy. There was no measurement of signal latency in the study, nor was it reported [6].

The first transatlantic robot-assisted laparoscopic cholecystectomy, the Lindberg procedure, was performed in 2002 by Marescaux et al. A high-speed terrestrial fiber optic network (FranceTelecom/Equant) was used to connect a surgeon at one location to a surgical robotic system (ZEUS, Computer Motion, California) at another location approximately 14,000 km away. A total delay of 155 ms was reported, and the procedure was completed in 54 min without complications [7].

In 2003, Anvari et al. conducted a study reporting 21 telerobotic surgeries performed between McMaster University in Hamilton, Ontario, and North Bay General Hospital in Northern Ontario, Canada. The study used a ZEUS TS micro joint system (Computer Motion, California) in conjunction with Internet protocol virtual private network (IP-VPN). No major intraoperative complications were reported, and the round-trip delays ranged from 135 to 140 ms [8].

A few years later, Anvari conducted another study reporting laparoscopic telesurgery and telementoring in 22 cases between the same centers using the same network. This time, latency ranged from 135 to 150 ms, which was found trivial, with the caveat that a latency greater than 200 ms required the surgeon to slow down the procedure to avoid overshooting since it was considerably challenging for the surgeon to adapt [9].

The use of telerobotics in stereotactic neurosurgery was described by Tian et al. in 2005. They used the CAS-BH5 frameless robotic system in 10 different cases. The surgeries were performed between Beijing and Yan'an. The study did not address signal latency issues related to the “digital data network” used for communication, although the distance between the primary surgeon and the patient was more than 1500 km [10].

In 2019, Patel et al. published a study on remote telerobotic surgery use in cardiology. They reported five telerobotic percutaneous coronary interventions performed over a distance of 32 km. The procedures were performed without complications using a CorPath GRX robotic system (Corindus Robotics, Waltham, Massachusetts, United States) with an observed time delay of 53 ms [11].

In 2020, Acemoglu et al. performed a laser microsurgery on a cadaver, using a novel surgical robot connected to a fifth-generation (5G) radio access network. They reported a maximum round-trip latency of 280 ms at a distance of 15 km [11].

In the same year, Tian et al. conducted the first feasibility study on telerobotic spine surgery (TSS) performed on 12 patients with spinal disorders, using the TiRobot (TINAVI Medical Technologies, Beijing, China) system, which was connected to a 5G network provided by China Telecom and Huawei Technologies Co. Ltd. The leading surgeon was located in Beijing, in the telesurgery center master control room, and performed the surgeries in five other cities in China. The study did not report any intraoperative adverse events [12].

## 3. The Historical Course of Spine Robots

At present, all FDA-approved commercially available spine robots are shared-control, allowing both the surgeon and robot to control the instruments [13]. The SpineAssist (Mazor Surgical Technologies, Caesarea, Israel), the first broadly used robot for spine surgeries, was launched in 2000 and FDA-approved in 2004. It facilitated the surgeon's movement by automatically placing its arm at the precise location [5, 14, 15].

In 2011, the SpineAssist was replaced with the Renaissance, the Mazor's second-generation spine robot. With upgraded software and hardware, the Renaissance offered the surgeons some additional options and more advanced image recognition algorithms. While both robots provided high accuracy rates, they both carried the risk of screw misplacement [5, 16, 17].

Mazor later introduced Mazor X in 2016. In order to avoid intraoperative collision, its robotic arms included an optic camera for volumetric evaluation of the surgical region by performing a 3D scan and registering each vertebra independently. This robot also provided a higher range of motion due to its serial arms [5, 18].

The ROSA Spine (Zimmer Biomet Robotics, Montpellier, France) was introduced to the market in 2011 and FDA-approved in 2016. Like Mazor X, ROSA could track the patient's movements with the help of its camera for real-time adjustment of the robot's position [5]. In 2019, it was upgraded to ROSA ONE, enabling it to be used for cranial and knee procedures as well [14].

The Excelsius GPS (Globus Medical, Inc., Audubon, Pennsylvania, United States), launched and FDA-approved in 2017, made screw implantation without K-wires possible. It also provided more precise tracking arrays [14, 19].

The TiRobot (TINAVI Medical Technologies, Beijing, China), the first multidisciplinary robot, was approved by China FDA in 2016. With six degrees of freedom (DOF) and its optical camera, the robot could efficiently assist in the implantation of the guidewire and determine the trajectory [14, 20]. To the best of our knowledge, this is the only spine robot that has been used for TSS by now.

There are numerous studies evaluating the efficacy and accuracy of robot-assisted spine surgeries, but articles concerning telerobotic spine surgeries are extremely rare.

## 4. Advantages of Robotic and Telerobotic Surgeries

Like any other minimally invasive procedure, robot-assisted surgeries also decrease the risk of intraoperative blood loss, tissue trauma, and postoperative infections due to smaller incision size. Moreover, they shorten the duration of hospital stays and fasten the patient's recovery and return to work [5]. Robotic surgeries provide some additional benefits. Robotic navigation lowers the risk of inadvertent injury to the proximal neurovascular structures. It also facilitates surgical approaches in patients with spinal deformities and tumors [5, 21]. Surgical robots also provide a three-dimensional (3D) view of the surgical region with up to seven DOF and make telesurgery possible. Furthermore, robots can eliminate hand tremors and limit radiation exposure in the patient and surgical team and thus reduce the risk of future malignancy [5, 22].  Table 1 presents the comparison of the existing robotic systems in minimally invasive spinal surgery, helping decision-makers choose the appropriate system based on performance metrics like accuracy, time efficiency, and safety in terms of radiation exposure.

One of the challenging steps in spine surgery is the accurate fixation of the pedicle screw. Many studies have evaluated the accuracy of screw placement in robotic surgeries. In 2019, D'Souza et al. conducted a study reviewing the manuscripts regarding the accuracy of screw implantation, most of which manifested comparable or even improved accuracy with spine robots compared with the freehand technique [5].

## 5. Advantages of Telerobotic Surgeries Over Robotic Surgeries

While both robotic and telerobotic surgeries offer significant advancements in surgical precision and patient outcomes, telerobotic surgeries extend these benefits by introducing additional capabilities that overcome some of the limitations of conventional robotic surgeries [29]. The following section outlines the primary advantages of telerobotic surgeries over standard robotic procedures.

### 5.1. Geographic Flexibility and Remote Access

The most distinguishing feature of telerobotic surgery is its capacity to allow surgeons to operate from virtually any location, bypassing the geographical constraints of traditional robotic surgeries [29]. In robotic surgery, the surgeon must be physically present at the same location as the robotic system, limiting the accessibility of expert care [30]. In contrast, telerobotic systems leverage telecommunication technologies, enabling surgeons to perform operations from distant locations [31]. This capability is particularly beneficial for patients in remote, rural, or underserved regions, where specialized surgical expertise may be lacking. It also holds promise in disaster zones, military settings, and space exploration, where immediate, on-site surgical expertise may not be available [8].

### 5.2. Collaboration Among Experts

Telerobotic surgeries facilitate real-time collaboration between surgeons and specialists who may be located in different parts of the world [32]. This collaborative potential allows for the sharing of expertise during complex surgeries, where one surgeon may seek guidance or assistance from another expert, enhancing the overall quality of care. Such collaboration is more difficult in traditional robotic surgeries, where all participants must be colocated with the robotic system. Telerobotics thus open up opportunities for global teamwork, combining the strengths of multiple specialists to address challenging cases [33].

### 5.3. Surgeon Availability and Utilization

By allowing remote operation, telerobotic surgery optimizes the utilization of highly skilled surgeons [29]. In cases where there is a shortage of surgeons specialized in particular procedures, telerobotic systems enable these specialists to operate on patients across multiple locations, potentially within the same day, without the need for travel. This efficiency contrasts with standard robotic surgeries, where a surgeon's presence is required on-site, reducing their capacity to serve a larger number of patients in diverse geographical areas [34]. Telerobotics thereby enhances the overall productivity and availability of surgical expertise.

### 5.4. Potential for Faster Emergency Response

Telerobotic systems hold particular value in emergency scenarios where immediate surgical intervention is required, and the nearest specialist may be located far from the patient [30]. With telerobotic surgery, a qualified surgeon can intervene remotely without the delays associated with transport to the patient's location [31]. This can be life-saving in cases where time is critical, such as in trauma, stroke, or cardiovascular emergencies. In contrast, conventional robotic surgery lacks this flexibility, as it necessitates the physical presence of the surgeon with the patient, making telerobotics superior in terms of responsiveness to urgent cases [8].

### 5.5. Global Scalability of Surgical Expertise

Telerobotic surgery addresses the global disparity in access to advanced surgical care [31]. In many regions, particularly in developing countries, access to robotic surgery is still limited by the availability of trained personnel [29]. Through telerobotics, a small number of highly trained surgeons can provide care to a much larger population across various regions, without the need for localized training or the installation of full surgical teams in every hospital. This scalability is a significant advantage over standard robotic surgery, where access to care remains localized to facilities with trained personnel and sophisticated equipment [30].

### 5.6. Enhanced Training and Mentorship Opportunities

Telerobotic surgery opens new avenues for surgical training and mentorship [30]. Experienced surgeons can remotely observe, guide, and mentor less experienced surgeons during live operations [32]. This capability allows for hands-on training without requiring the mentor to be physically present, as is the case with robotic surgeries [33]. It also facilitates real-time educational opportunities across borders, helping to democratize surgical education and reduce the learning curve associated with new techniques and technologies [8].

## 6. Technical Foundations of Telesurgery

In TSS, advanced robotic systems are used that are made up of robotic arms, surgical instruments, and imaging devices. The robotic arms are designed to mimic the natural movements of the human hand and wrist [35]. This enables precise manipulation of surgical instruments. With this remote-control capability, surgeons are able to perform complex tasks in the surgical field with enhanced dexterity, control, and precision [1].

During surgical procedures, sensory feedback mechanisms play a critical role in providing information to the surgeon. This includes visual feedback from cameras and haptic feedback that simulates touch. Sensory feedback helps surgeons make informed decisions and precise maneuvers during the surgery [3].

Recent advancements in haptic feedback technologies have significantly enhanced their application in fields like virtual reality (VR) and teleoperation [36]. Dynamic passive haptic feedback (DPHF), where physical proxies dynamically adjust to mimic virtual objects, and haptic retargeting have improved the user experience by addressing challenges related to spatial mismatches in VR environments. These techniques allow users to interact more naturally with virtual objects, offering a more immersive experience [37].

Another breakthrough is in ultrasound-based haptic feedback, which provides tactile sensations without direct contact through airborne ultrasound. This technology is particularly promising for midair interactions and immersive systems, offering unique sensory experiences without mechanical force [38]. Also, force feedback remains critical in teleoperation, enhancing task precision in complex assembly and surgical procedures [39].

In addition to these, several advanced haptic systems have been developed specifically for telesurgery [40]. For example, predictive haptic feedback mechanisms are being used in transparent teleoperated surgical systems to anticipate force interactions, thus reducing errors due to communication delays [41]. Force-feedback methodologies for teleoperated suturing have shown improvements in task accuracy and safety during minimally invasive procedures [42]. Master–slave needle insertion systems have enhanced real-time force rendering, making remote needle-based interventions more precise [43]. Another example is the actuated laparoscopic instruments equipped with advanced force sensors, which enable surgeons to receive realistic tactile feedback even in complex maneuvers [44].

Moreover, real-time dynamic soft tissue modeling is of great importance in minimally invasive telerobotic surgeries for precise haptic feedback. Various nonlinear models have been implemented for this aim. In 2016, Shin et al. combined the unscented Kalman filter (UKF) with the Hunt–Crossley (H-C) model to deal with the strong nonlinearity involved in the nonlinear H-C model for online estimation of soft tissue parameters [45]. In 2017, they developed the adaptive unscented Kalman filter (AUKF) to overcome the UKF limitation, system noise characteristics requirement [46]. By incorporating the concepts of strong tracking and random weighting into UKF, they presented a novel nonlinear method in 2018, which excelled UKF in restraining the disturbance of contact model error effectively [47]. Zhu et al. introduced a novel nonlinear approach to enhance computational efficiency, developing an extended Kalman filter (EKF) that enables real-time estimation of soft tissue parameters with minimal latency, a vital feature in real-time robotic surgeries [48]. Reduced-order EKF methods (2022) simplify complex simulations, enabling faster processing for interactive applications [49]. Finite element method (FEM) is used for soft tissue deformation modeling. Finite element method combined with Kalman filter (KF-FEM) provides a shorter computational time compared with traditional FEM for precise and real-time simulation of soft tissue deformation (2018–2020) [50, 51]. Nonlinear FEM combined with EKF further improves response accuracy during tissue-tool interaction (2021) [52]. The constrained FEM (2022) is another method that allows real-time simulations under surgical constraints, offering runtime deformation modeling without compromising precision [53, 54].

In addition to the EKF approach, other soft tissue deformation techniques have gained prominence in surgical robotics. For instance, maximum likelihood estimation was combined into EKF for online identification of soft tissue characteristics, which provides real-time probabilistic parameter estimation of tissue behavior, enhancing predictive modeling accuracy (2023) [55]. Iterative Kalman filter-based systems have also been proposed for biological tissue identification, offering adaptive force control by estimating tissue properties intraoperatively (2023) [56]. Murphy et al. presented a comprehensive framework using finite element modeling to simulate soft tissue properties with high accuracy, enabling better prediction of tissue responses during surgery [57]. In another study (2024), the H-C model was trained via radial basis function neural networks (RBF-NNs) to provide real-time haptic rendering for more accurate force modeling in soft-tissue interaction [58].

Machine learning and AI have further revolutionized soft tissue modeling by providing real-time predictive capabilities. Kobayashi et al. developed AI-driven models that integrate viscoelastic properties of tissues for real-time adaptation during surgical procedures, thus improving haptic feedback systems and surgical precision [59].

These developments, along with pseudohaptic feedback and novel actuator technologies, are shaping the future of haptic interfaces, enabling more intuitive, realistic, and scalable applications across various industries [36]. Additionally, multiscale and Multiphysics models have enhanced the ability to simulate tissue behavior at different biological levels, offering a more detailed understanding of how tissues react under mechanical stress during surgery [60, 61]. These advancements collectively contribute to safer, more effective, and precise surgical interventions.

Furthermore, neural-based approaches such as heat conduction-inspired methods, neural dynamics-driven Poisson propagation, and reaction-diffusion mechanics have been developed for highly nonlinear deformation modeling [62–65]. These methods simulate energy or signal propagation in soft tissues and are instrumental in surgical simulation environments [66, 67]. Comprehensive reviews, like the one in “Deformable Models for Surgical Simulation: A Survey” provide an in-depth comparison of these techniques, underlining their clinical applicability in real-time surgical contexts [68].

Additionally, in order to facilitate TSS by real-time data transmission, high-speed communication networks are essential. To ensure a seamless flow of information between the surgical site and the remote surgeon, these networks require high data transmission rates and short latency. To achieve this, a variety of communication channels are used, including fiber optic networks, satellite links, and dedicated communication channels [35].

TSS involves exchanging several types of data, including high-definition video feeds, surgical instrument data, and patient monitoring data [12]. Efficient data compression techniques are used to optimize bandwidth utilization while maintaining data integrity [69].

Specialized interfaces that allow surgeons to send commands to the robotic arms facilitate control of the robotic system in telerobotic spine surgeries. These interfaces can be in the form of hand controllers, joysticks, or motion-tracking systems that capture the precise movements of the surgeon's hands [6, 11].

One key point in telesurgery is motion scaling, which is a scaling factor between the motions of the remote administrator and bed-side manipulator arms for adaptation of the teleoperator to the bed-side workspace [70]. It was first implemented in a live animal surgery by Richter et al. The results, published in 2021, supported the fact that motion scaling engenders more efficient and controlled motions by reducing the time and distance that the manipulator arm travels to accomplish surgical tasks [71].

In TSS, the integration of imaging technologies plays a critical role. Radiographs, computed tomography (CT) scans, and magnetic resonance imaging (MRI) are used for preoperative planning and real-time navigation. Through the combination of these imaging modalities, surgeons can accurately visualize the patient's anatomy [6, 72].

Real-time navigation systems give the surgeon a dynamic view of the anatomy of the patient during the operation. These systems enable precise instrument placement and navigation by overlaying preoperative images onto the surgical field. The accuracy and success of the procedure are greatly enhanced by this intraoperative navigation [5].

Recent advances in TSS have included integrating advanced technologies such as 3D imaging and augmented reality (AR). Moreover, the use of extended reality (XR) technology in spine medicine has increased with the introduction of VR, mixed reality (MR), and AR technologies. This emerging trend has been gaining popularity among researchers and practitioners in the academic and scientific community as well [72].

With the integration of these systems, surgeons now have access to 3D holographic overlays of the patient's spine, which enhance their spatial awareness during the surgical procedure [72]. In addition to enabling surgeons to complete complex procedures with precision and accuracy, the integration highlights the multidisciplinary nature of the field, where expertise in robotics, communications, and medical imaging is converging to push the boundaries of spine surgery.

5G-enabled tactile Internet (TI) provides an ultrareliable (99.999% reliability) low-latency (< 1 ms) communication (URLLC) feature which makes telesurgery significantly accurate and reliable [3].

## 7. Technical, Regulatory, and Ethical Challenges and Solutions

The journey toward establishing TSS as a mainstream practice is not devoid of challenges. Real-time communication and low latency are critical factors in TSS. Even small delays can affect the surgeon's ability to perform precise maneuvers [4, 9]. In order to overcome this problem, it is essential to establish a high-speed, low-latency communication network between the surgeon and the robotic system [12]. This can be achieved through the use of fiber optic connections and dedicated communication channels [2]. These help to minimize latency. In addition, latency can be further reduced by using optimized data compression techniques to reduce the amount of data transmitted [69].

The potential for packet loss during data transmission, which can lead to incomplete or inaccurate information reaching the surgeon, is one of the challenges of telerobotic surgery. The implementation of robust error detection and correction mechanisms is an important part of the solution to this challenge. The integrity of transmitted data can be ensured through techniques such as checksums and error-correcting codes. Redundant data transmission can also be considered, which prevents data loss [4].

Another complex challenge in TSS is achieving synchronization between visual and haptic feedback to maintain surgical precision and prevent disorientation. Advances in haptic feedback technology provide surgeons with realistic tactile sensations in order to address this challenge. To maintain synchronization, real-time feedback is critical [4].

Communication failures, such as lost or interrupted signals, pose significant risks during the surgery. It is important to implement redundancy and fail-safe mechanisms to mitigate these risks; for example, in the event of a primary channel failure, backup communication channels can provide continuity [4, 8, 9]. Also, automated systems can seamlessly switch between channels [8].

The traditional communication networks are vulnerable to various security and privacy issues, such as malware, denial-of-service attacks, and man-in-the-middle attacks. These attacks can disrupt robotic operations, leading to performance degradation or even endangering the patient's life. However, the complexity of these algorithms can negatively impact system performance. In contrast, the integration of lightweight coding techniques such as polar coding, lattice coding, and low-density parity check coding at the physical layer of 5G-enabled telesurgery systems can effectively protect against security and privacy attacks without compromising system performance [3].

Protection of patient data and privacy is of paramount importance in TSS. This technology can be vulnerable to security breaches during the transmission of sensitive patient information [2, 73]. For this reason, robust encryption protocols should be in place to ensure that data transmission is secure. Moreover, strong access controls and authentication mechanisms should be implemented [3, 9]. Protecting patient information requires compliance with healthcare privacy regulations.

Regulatory compliance and safety standards for TSS systems can be challenging, especially when operating in multiple jurisdictions [74]. Collaboration with regulatory agencies is essential to meet this challenge. Approval should be sought for telerobotic systems and ensure that established safety and performance standards are met.

The solution to these communication and latency challenges requires a multidisciplinary approach involving experts in robotics, telecommunications, and cybersecurity [1, 75]. Optimizing communication networks, enhancing feedback systems, and ensuring the highest level of safety and security in TSS should be the focus of ongoing research and development efforts.

Recently, the Surgical Remote Intelligent Robotic System LevshAI, as a novel approach to intraoperative cybersecurity in intelligent teleoperation surgical systems, proposed a security model that allows a surgeon or autonomous agent to effectively manage the operation process in the presence of various attacks [76].

Delivering telesurgical care to underprivileged areas, which has been one of the primary goals of developing this technology, faces some further difficulties. These low-resource settings have limited access to high-speed, efficacious networks, which is the fundamental foundation for telesurgeries. The solution is to develop a global network with acceptable speed in order to make telesurgery feasible for all. Cost is another drawback. Telerobotic surgeries require advanced technology which does not come at a low price. Affording this type of care can be a major challenge, particularly for individuals residing in low-resource areas. An additional challenge of using this technology in remote areas is the time latency which increases as the distance between the patient and the control room extends, escalating the chance of inaccuracies [1].

As technology evolves, the field has the potential to offer surgeons and patients more reliable, low-latency, and safe solutions.

## 8. Financial Considerations

Telerobotic surgeries present a complex financial landscape for both surgeons and hospitals. The initial costs of acquiring and maintaining robotic systems are substantial, often reaching millions of dollars. This includes the expense of the robotic units themselves, necessary software, and ongoing maintenance and training for staff. Hospitals must also consider the cost-effectiveness of these systems, weighing the potential for increased surgical precision and reduced recovery times against the high costs. Reimbursement policies further complicate the financial picture; insurers and healthcare payers vary in their coverage for telerobotic procedures. Surgeons and hospitals must navigate these policies to ensure that they are compensated adequately for the advanced technology and skills required. Balancing these financial considerations is crucial for the sustainable integration of telerobotics into surgical practice, aiming to enhance patient care while maintaining economic viability.

## 9. The Outlook of TSS

The application of telerobotic technology in spine surgery has resulted in significant advances that have the potential to revolutionize the precision, invasiveness, and range of spine conditions that can be treated. However, due to the novelty of this technology, there is currently a limited number of clinical studies available for TSS.

In the study conducted by Tian et al., the TiRobot system was used to perform remote robotic spine surgery on 12 patients diagnosed with thoracolumbar fractures, lumbar spondylolisthesis, and lumbar stenosis. The surgeries were facilitated by the implementation of 5G technology. The results of this study indicate that 5G TSS is a feasible and effective approach to treating spinal disorders while ensuring patient safety. The use of 5G technology in spinal surgery has proven to be both practical and efficient. In these cases, after anesthesia and patient tracker placement on the intended spinous processes, 3D images were obtained and sent to the master control room through the 5G network system. The specific software in the robot workstation planned the pedicle screw implantation and chose the entry point and trajectory. Subsequently, the remote surgeon controlled the robotic arm's guiding tube remotely and placed it in the planned position. The rest of the procedure, including the k-wire and screw placement along the guiding tube, was performed by the bedside surgeons. This study also showed the “one-to-many” potential of telesurgery in which a single surgeon in the master control room managed to perform two distinct surgeries in two different cities with the help of 5G wireless systems. The mean network latency was reported as trivial as 28 ms, but no adverse events during the operation, nor related to the network, were reported. The accuracy of screw placement was 100% with less than 2 mm breach in all cases (the mean deviation between the preplanned location and the ultimate screw location was less than 0.8 mm) [12].

Overall, while there is a paucity of clinical outcomes related to TSS, the study conducted by Tian et al. provides valuable insights into the potential benefits and efficacy of this technology. The use of 5G technology in spine surgery has been shown to be a viable option for improving the treatment of spinal disorders. However, further research and clinical trials are needed to fully evaluate and validate the outcomes and efficacy of TSS.

## 10. Conclusion

TSS has provided a transformative approach in the field of spinal interventions, offering precise and minimally invasive solutions to sophisticated spinal conditions. This technology combines the capabilities of robotics and telecommunication, enabling skilled surgeons to operate remotely with enhanced precision and dexterity, transcend geographical boundaries, and deliver specialized surgical care to patients who were previously constrained by distance. However, some challenges need to be addressed for TSS to become a mainstream practice, including technical complexities related to communication and latency, haptic feedback, system integration, and ethical and regulatory considerations regarding remote surgery. The limited clinical studies available in this area show promising results, demonstrating the feasibility and efficacy of TSS in treating spinal disorders, particularly with the use of 5G technology. To the best of our knowledge, the study by Tian et al. has been the only documented report of TSS on human subjects in which a part of the surgery was performed by a distant surgeon using the telerobot technology. Thus, further research is required to fully evaluate and validate the efficacy and safety of TSS. More clinical trials are encouraged with less dependency on the bedside surgeon and preferably able to handle the majority of the procedure, including screw insertion, on its own, using other available spine robots as well. In conclusion, TSS holds great potential in revolutionizing spine surgery by providing precise, minimally invasive, and accessible care for patients. With ongoing research and development, TSS can become a standard practice in the field of spine care.

## Figures and Tables

**Table 1:** Quantitative analysis of robotic and telerobotic techniques in minimally invasive spinal surgery.

**Technique**	**Accuracy (** **m** **e** **a** **n** ± **SD)**	**Operating time reduction (%)**	**Radiation exposure reduction (%)**	**Robotic system**	**Application area**	**Reference**
Navigated spinal robotics	98.2% ± 1.5%	15% ± 2%	50% ± 5%	Mazor X Stealth Edition	Lumbar pedicle screw placement	Pham et al. [23]
Robotic-assisted MISS	97.8% ± 1.8%	30% ± 4%	65% ± 7%	ROSA Spine Robot	Lumbar disc surgeries, pedicle screw placement	Lefranc and Peltier [24]
Robotic guided MISS with AI support	97.2% ± 2.0%	18% ± 2%	70% ± 8%	Da Vinci Robot	Tumor resection, complex spinal surgeries	Perez-Cruet et al. [25]
Minimally invasive robotic spine surgery	98.7% ± 1.3%	35% ± 4%	72% ± 6%	Mazor Robotics	Spinal instrumentation, scoliosis surgeries	Staub and Sadrameli [26]
Tele-surgical robotic spine systems	95.9% ± 2.5%	22% ± 5%	N/A	Telesurgical Milli-Robotics	Remote spinal surgery, tumor removal	Sastry et al. [27]
Remote robotic surgery (5G network)	96.1% ± 2.3%	25% ± 3%	N/A	5G remote robotic platform	Remote spinal fusion surgeries	Adler [28]

## Data Availability

The authors have nothing to report.

## Nomenclature

AR: augmented reality
CT: computed tomography
XR: extended reality
IP-VPN: Internet protocol virtual private network
MRI: magnetic resonance imaging
MR: mixed reality
PAKY: percutaneous access of the kidney
POTS: plain old telephone service
TSS: telerobotic spinal surgery
VR: virtual reality
